# 离线全二维逆流色谱-液相色谱分离莪术油成分

**DOI:** 10.3724/SP.J.1123.2023.04008

**Published:** 2023-12-08

**Authors:** Xin TONG, Yang JIN, Jing JIN, Ping LIU, Chunyan WU, Shengqiang TONG

**Affiliations:** 1.台州科技职业学院,浙江台州318020; 1. Taizhou Vocational College of Science & Technology, Taizhou 318020, China; 2.浙江工业大学,浙江杭州310014; 2. Zhejiang University of Technology, Hangzhou 310014, China

**Keywords:** 逆流色谱, 液相色谱, 全二维色谱, 正交性, 莪术油, countercurrent chromatography (CCC), liquid chromatography (LC), comprehensive two-dimensional chromatography, orthogonality, *Curcuma* volatile oil

## Abstract

中药挥发油成分复杂,一维色谱分离由于有限的峰容量难以完全分离中药挥发油成分,全二维气相色谱为分离挥发油成分提供了有力的方法,然而气相色谱一般无法用于天然活性成分的筛选。为建立挥发油成分全二维色谱分析新方法,研究建立以液相色谱为基础的全二维色谱分离分析方法。本文主要研究全二维逆流色谱-液相色谱分离莪术油成分的方法,并探讨两种色谱技术之间的正交性,为活性成分筛选提供新的技术支持。通过优化离线全二维逆流色谱-液相色谱分离方法,对全二维色谱峰容量、正交性和空间覆盖率进行度量。优化液相色谱分析条件并筛选逆流色谱分离两相溶剂体系,通过比色法筛选了逆流色谱两相溶剂体系并采用下相为流动相进行梯度洗脱。在290~375 min采用推挤洗脱,莪术油在第一维逆流色谱分离中达到了良好的分离。第二维反相高效液相色谱的流动相组成为乙腈(A)和水(B)。梯度洗脱程序为0~10 min, 50%A~65%A; 10~14 min, 65%A; 14~21 min, 65%A~85%A; 21~25 min, 85%A~95%A; 25~30 min, 95%A~55%A; 30~40 min, 55%A。在上述条件下进行全二维色谱分析,结果表明该全二维分离体系总峰容量达954个,理论峰容量达到一维色谱的10倍以上,同时具有良好的正交性(线性相关性*r*=0.17)和空间覆盖率(68.1%)。以上结果表明,离线全二维逆流色谱-液相色谱分离莪术油具有良好的正交性,能显著提高复杂样品的分离效果,为挥发油物质基础研究和活性成分筛选以及二维色谱指纹图谱的建立提供了新的方法与思路。

## Abstract (en)

The chemical constituents of volatile oils used in traditional Chinese medicine are highly complex. Thus, achieving the complete separation of volatile oils by one-dimensional chromatography is difficult owing to the low peak capacity of the technique. Although comprehensive two-dimensional gas chromatography provides an efficient means for separating volatile oils, it cannot be used to screen bioactive components because of its limitations. Therefore, developing a new method to separate volatile oils based on liquid chromatography is of great significance in efforts to obtain new approaches to screen bioactive components in volatile oil. The objectives of the present study are to establish an efficient method for separating the chemical constituents of *Curcuma* volatile oil using off-line comprehensive two-dimensional countercurrent chromatography-liquid chromatography (CCC-LC) and to investigate the two-dimensional peak capacity, orthogonality, and spatial coverage of this method. Both CCC and LC conditions were optimized. A biphasic *n*-hexane-methanol-water solvent system was selected via the colorimetric method, and the lower phase was used as the mobile phase in gradient-elution mode: 0-55 min, *n*-hexane-methanol-water (5∶2∶3 v/v/v); 55-170 min, *n*-hexane-methanol-water (5∶3∶2, v/v/v); 170-290 min, *n*-hexane-methanol-water (5∶4∶1, v/v/v). After gradient elution, elution-extrusion elution mode was applied within 290-375 min. Good resolution was achieved by the CCC separation process. The HPLC separation process was carried out with gradient elution using a mobile phase composed of acetonitrile (A)-water (B): 0-10 min, 50%A-65%A; 10-14 min, 65%A; 14-21 min, 65%A-85%A; 21-25 min, 85%A-95%A; 25-30 min, 95%A-55%A; 30-40 min, 55%A. *Curcuma* volatile oil was separated under the above optimized two-dimensional separation conditions, and the data obtained were drawn into a two-dimensional contour map using Matlab software. The calculated total peak capacity exceeded 954, which was 10 times more than that of one-dimensional chromatography. High orthogonality (*r*=0.17) and spatial coverage factor (68.1%) were also obtained. Our research provides a new methodology for separating volatile oils used in traditional Chinese medicine as well as an approach for evaluating the quality of traditional Chinese medicinal herbs using two-dimensional chromatographic fingerprints.

从天然产物中筛选药用活性成分是新药研究与发现的重要途径,色谱分离技术对活性组分的筛选起到至关重要的作用^[1]^。中药挥发油往往含有多种活性成分,然而由于其成分复杂,一维色谱有限的峰容量难以完全分离挥发油组成。尽管毛细管气相色谱与全二维气相色谱为挥发油成分分析提供了有力手段,但是气相色谱分离后因为组分难以回收利用,因此不适用于活性成分的筛选。发展新型的以液相色谱为基础的多维色谱分离新方法,对于中药挥发油复杂体系分离分析及活性成分筛选均具有重要的意义。

全二维色谱技术结合多种分离机制或分离模式实现样品分离,特别适用于物质极性相似、性质相近与结构相似组分的分离,可显著提高峰容量、选择性和分离度^[2,3]^。目前全二维色谱正交性研究集中于传统气相色谱与液相色谱^[4,5]^。在传统的二维液相色谱与气相色谱中,选用不同分离机制的色谱柱能显著增加两个维度的正交性与峰容量,从而提高分离效率,但仍存在一些难以避免的缺陷:样品的不可逆吸附性导致样品无法完全进入第二个维度进行分析;两个维度之间由于不同的分离色谱柱,如正相与反相,导致流动相难以兼容。为了充分发挥二维色谱的分离潜力,从实质上提高二维分辨率,准确、高效地对复杂组分进行分离分析,构建高正交性的色谱分离体系已经成为多维色谱研究的热点^[6,7]^。正交性与峰容量是衡量多维色谱在分离能力与分离机制独立性水平的重要参数^[8]^。

逆流色谱(countercurrent chromatography, CCC)是近年来兴起的高效分配色谱技术,其主要特征为流动相与固定相均为液体,因此固定相无需固体载体,运行与维护成本相对较低且容易实现样品的制备性分离。与传统液相色谱相比,具有以下几个优势:①没有不可逆吸附发生;②对进样样品要求低,无需复杂预处理;③样品进样量大,常规进样量可达数百毫克至克级。因此逆流色谱技术与传统液相色谱分离技术理论上具有良好的互补性。然而,以这两种技术为基础的全二维色谱研究报道不多,仅有少量文献应用于石油化工、中药挥发油的分离分析中^[9⇓-11]^。笔者所在课题组采用全二维逆流色谱-液相色谱对中药虎杖醇提物、三七皂苷等天然产物进行了分离分析,结果表明两种色谱技术即使均在反相洗脱条件下,仍然具有良好的互补性^[12⇓-14]^。目前尚未见对全二维逆流色谱-液相色谱分离中药挥发油的正交性进行相关研究的报道。

莪术为姜科植物蓬莪术*Curcuma phaeocaulis* Val.、广西莪术*Curcuma kwangsiensis* S. G. Lee et C. F. Liang或温郁金*Curcuma wenyujin* Y. H. Chen et C. Ling的干燥根茎。莪术主要用于癥瘕痞块,瘀血经闭,胸痹心痛,食积胀痛。《中国药典》记载莪术根茎中挥发油不得少于1.5%(mL/g),主要为倍半萜类和单萜类化合物,具有抗氧化、抗肿瘤、抗炎、抗菌、抗病毒等药理作用^[15]^。由于挥发油成分复杂,常规分离分析方法无法完全分离其组成,研究针对挥发油的新型分离分析方法对于阐明挥发油药效物质基础、筛选活性成分、质量控制及进一步开发利用都具有重要意义。

## 1 实验部分

### 1.1 仪器与试剂

高效液相色谱仪(LC-20A,岛津企业管理(中国)有限公司);半制备型高速逆流色谱仪(TBE-200V,上海同田生物技术有限公司);柱塞式恒流泵(s-1007,北京圣益通技术有限公司);恒温循环器(HX-1050,宁波新芝生物科技有限公司);色谱3000工作站(Sepu3000,杭州普惠科技有限公司);旋转蒸发仪(BUCHI R-300,瑞士步琦公司);自动部分收集器(BSZ-160,上海嘉鹏科技有限公司)。

莪术油购自江西省吉水县顺民药用香料油提炼厂;甲醇、乙腈为色谱纯(美国Fisher Scientific公司),液相色谱用水为实验室重蒸水,其他试剂均为分析纯(国药集团化学试剂有限公司)。

### 1.2 液相色谱条件

色谱柱为岛津Shim-pack GIS C_18_分析柱(250 mm×4.6 mm, 5 μm);柱温30 ℃;流动相A为乙腈,B为水。梯度洗脱条件:0~10 min, 50%A~65%A; 10~14 min, 65%A; 14~21 min, 65%A~85%A; 21~25 min, 85%A~95%A; 25~30 min, 95%A~55%A; 30~40 min, 55%A。流量1 mL/min;进样量20 μL;检测波长215 nm。

### 1.3 样品溶液配制

精密量取莪术油10.0 mg溶于50 mL容量瓶中,用甲醇-水(70∶30, v/v)稀释至刻度。

### 1.4 逆流色谱条件

逆流色谱分离均为头-尾洗脱模式,溶剂体系为正己烷-甲醇-水(5∶4∶1, v/v/v),充分混匀后在分液漏斗中静置分层,待两相平衡后,将两相分别放入试剂瓶内,超声脱气15 min。将上相泵入逆流色谱分离柱内作为固定相,调节分离柱转速至800 r/min,待转速稳定后,将溶剂体系的下相作为逆流色谱流动相,以流量2 mL/min泵入柱内。当分离柱内两相平衡后,采用六通阀注入莪术油50 mg,样品用各5 mL上相和下相超声溶解,作为进样样品。紫外检测波长为214 nm,采用自动部分收集器收集洗脱组分,每1 min收集一个洗脱组分。

### 1.5 数据处理

液相色谱数据通过岛津Labsolution软件导出,逆流色谱数据通过Sepu3000工作站导出,全二维色谱分离莪术油的等高线图通过Matlab绘制。

## 2 结果与讨论

### 2.1 分离条件优化

#### 2.1.1 液相色谱条件的优化

建立并优化反相高效液相色谱分离莪术油成分的方法,对流动相组成、流动相比例、分析时间以及检测波长进行了考察。

实验表明当采用甲醇作为流动相的有机相时并不能有效分离莪术油化学成分,有机相更改为乙腈后,在峰形、峰数和分离度方面,都有较为明显的改观。因此,选择流动相为乙腈-水体系。

同时,考虑到离线全二维色谱分析时间的要求,对液相色谱分析时间设定在30 min。莪术油主要化学成分为倍半萜类和单萜类化合物,包括*β*-榄香烯、吉马酮、莪术呋喃二烯酮、莪术二酮、莪术烯、*α*-蒎烯、莰烯、*β*-蒎烯等,大多数成分含有双键,因此检测波长设定为215 nm。图1为莪术油的反相高效液相色谱图。

**图1:**
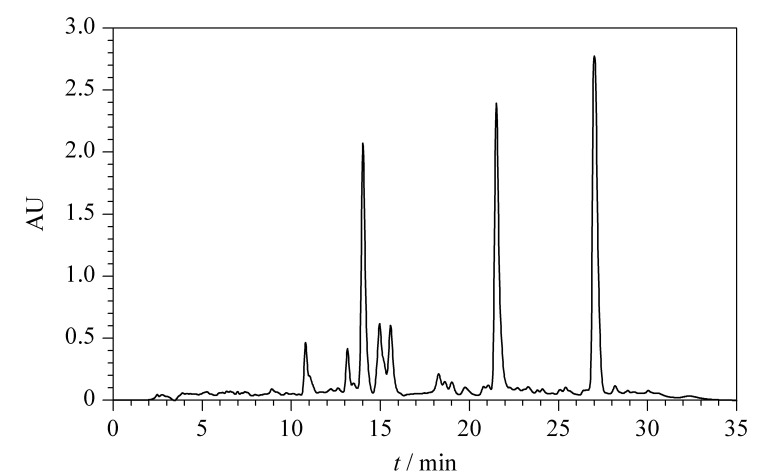
莪术油反相高效液相色谱色谱图

#### 2.1.2 逆流色谱条件的优化

逆流色谱属于液液分配色谱,其分离条件的选择与传统液相色谱有较大区别,一般认为逆流色谱分离条件的优化主要取决于两相溶剂体系的筛选以及洗脱模式的确定。逆流色谱两相溶剂体系的筛选主要考虑:溶剂体系对样品的溶解度大小;两相溶剂体系分层时间一般不超过30 s;目标组分在两相溶剂体系中的分配系数应为0.2~5.0。分配系数太大,保留时间较长,色谱峰展宽变大;分配系数过小,则保留时间很短,样品中目标组分容易与流动相前沿一同洗脱出来从而导致分离度较差。因此,在传统的逆流色谱分离天然产物、合成产物以及发酵产物等混合物时,目标组分分配系数的测定非常重要,通常采用液相色谱、气相色谱或薄层色谱用于测定混合物样品中目标组分在两相中的分配系数,分别测定目标组分在溶剂体系上相和下相中的浓度,根据浓度的比值计算分配系数,或者在薄层色谱上判断分配情况,直至筛选到合适的溶剂体系用于目标组分的分离。针对混合物中目标组分的分离,关于逆流色谱溶剂体系的选择已有较多的文献和著作进行了系统介绍^[16⇓⇓-19]^。

由于本文主要探讨逆流色谱反相洗脱模式下与传统反相高效液相色谱分离天然产物成分的互补性,并非分离纯化莪术油中的目标成分,本研究中溶剂体系的筛选需要考虑莪术油中整体化学成分的分配情况。由于莪术油为淡黄色液体,因此我们采用了简单易行的比色法来筛选溶剂体系,观察莪术油在两相体系中的分布情况来筛选两相体系。中药挥发油大多数成分为小极性组分,因此主要考察了小极性溶剂体系。分离挥发油的常用溶剂体系为正庚烷-乙酸乙酯-乙腈体系、正己烷-乙醇-乙腈体系、正己烷-乙腈-水体系和正己烷-甲醇-水体系等。通过比色法,发现正己烷-乙腈-水(5∶2∶3, v/v/v)和正己烷-甲醇-水(5∶3∶2或5∶4∶1, v/v/v)可以达到理想的样品分配效果,考虑到试剂环保性与经济性,重点考察了正己烷-甲醇-水体系。由图2可见,莪术油在正己烷-甲醇-水(5∶4∶1, v/v/v)体系中两相能够均匀溶解,呈现接近的颜色。因此,选择正己烷-甲醇-水(5∶4∶1, v/v/v)作为分离挥发油的两相溶剂体系,其中水相作为流动相,有机相作为固定相。

**图2:**
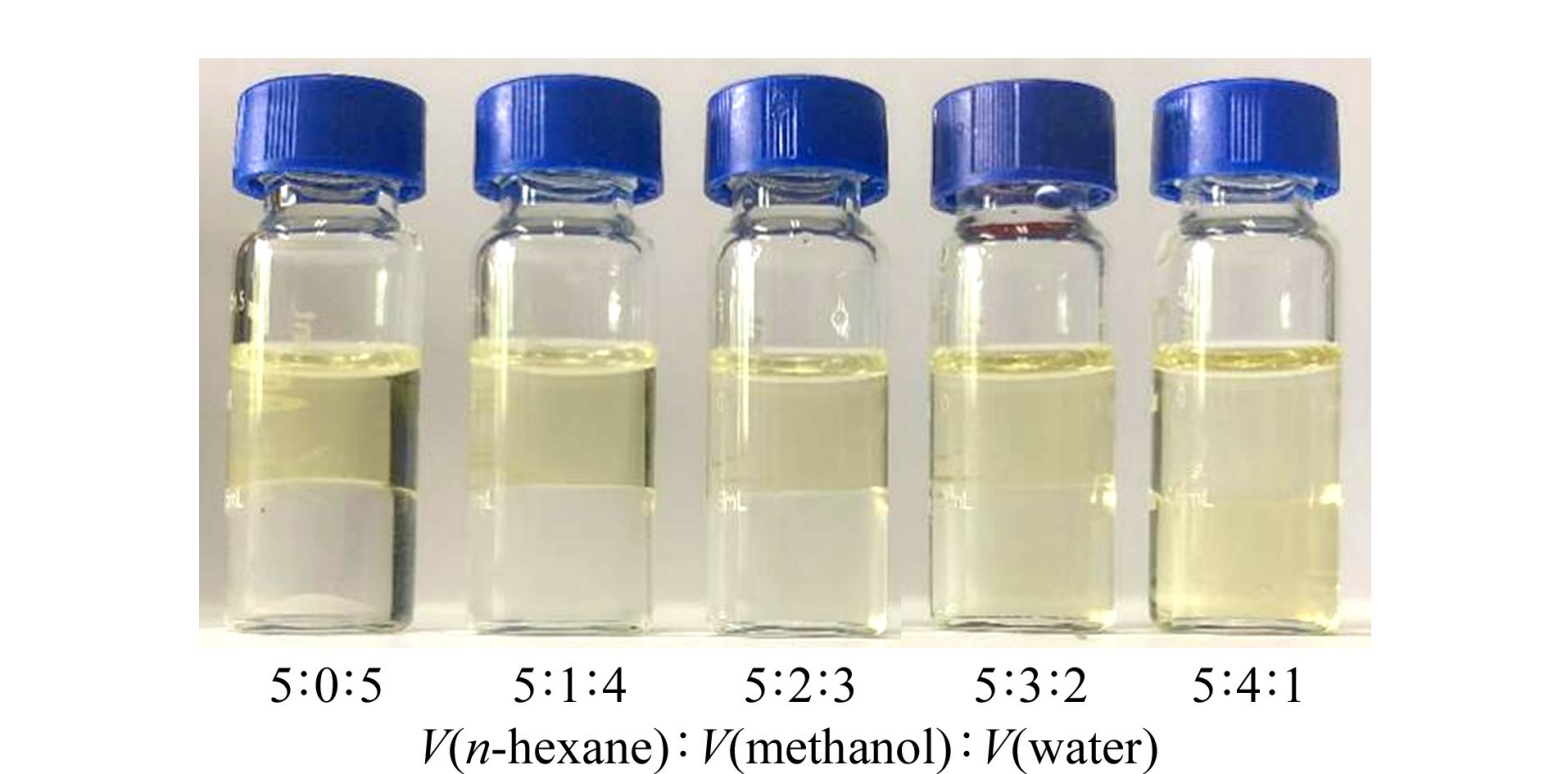
比色法筛选两相溶剂体系

根据上述筛选的两相溶剂体系正己烷-甲醇-水(5∶4∶1, v/v/v),采用逆流色谱分离莪术油成分,上相为固定相,下相为流动相,实验结果见图3a,其中保留时间225 min开始为洗脱推挤模式。从图中可以看出,莪术油主要化学成分主要集中在130 min以内,未能得到理想的分离与均匀分散,与液相色谱类似,等度洗脱条件下组分无法获取良好的分离。因此,实验进一步考察了梯度洗脱的方法,将水相中甲醇含量逐步提升。梯度洗脱条件:0~55 min,正己烷-甲醇-水(5∶2∶3, v/v/v); 55~170 min,正己烷-甲醇-水(5∶3∶2, v/v/v); 170~290 min,正己烷-甲醇-水(5∶4∶1, v/v/v)。290~375 min,采用推挤洗脱。推挤洗脱过程中流速为5 mL/min,固定相保留率为57.9%。结果表明,挥发油组分的分离有了明显改善,且均匀分散在一定的保留时间内,见图3b。

**图3:**
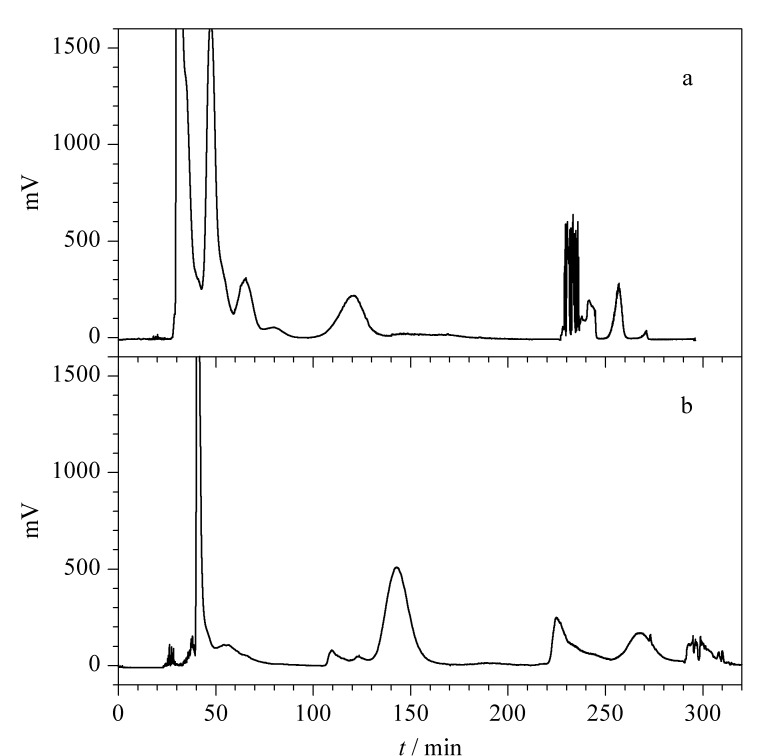
莪术油在(a)等度洗脱和(b)梯度洗脱模式下的逆流色谱图

### 2.2 全二维逆流色谱-液相色谱图谱构建

在第一维逆流色谱梯度洗脱分离过程中,从逆流色谱保留时间25 min开始至310 min内每1 min收集一个样品,共收集得到285个逆流洗脱组分。每一个组分进行液相色谱分析,以液相色谱数据为基础进行Matlab作图,得到全二维色谱的化学成分等高线图(图4)。从图中可以看出,莪术油中主要成分都得到了良好分离,且两个维度的色谱分离构成了良好的互补性,含量高的几个主要成分在液相色谱中未能良好分离,但是在逆流色谱中得到了很好的分离;同样在逆流色谱无法分离的组分,在液相色谱中具有良好的分离度,譬如图中组分A和B以及组分C和D。A和B两个组分在HPLC中同时洗脱,洗脱时间为12.1 min,而在逆流色谱分离中A和B的保留时间分别为100 min和140 min,得到完全分离;在逆流色谱分离过程中,洗脱时间为170~195 min时,C和D两个化合物同时洗脱,但在HPLC中分离获得保留时间为14.5 min和17.4 min的化合物C和D。逆流色谱分离主要依据溶质分配系数的差异,而液相色谱兼具有固液吸附与液液分配保留机理,因此反相液相色谱与反相逆流色谱存在良好的互补性,从正交性角度进行二维色谱的分离,可以显著提高样品分离度。

**图4:**
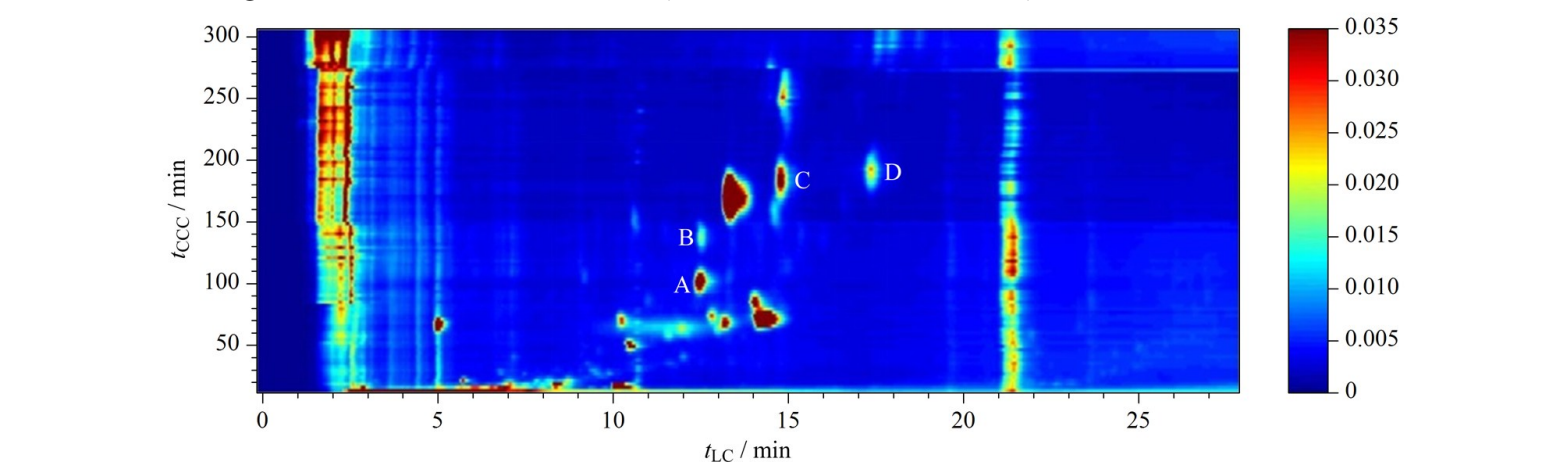
离线全二维逆流色谱-液相色谱分离莪术油的二维等高线图

### 2.3 全二维色谱分离莪术油正交性分析

#### 2.3.1 峰容量

根据Giddings多维色谱峰容量计算方法,梯度洗脱条件下采用公式(1)对全二维逆流色谱-液相色谱分离莪术油成分的峰容量进行计算^[20]^:


(1) $n_\mathrm{c}=1+\frac{t_\mathrm{g}}{1/n\sum_\mathrm{l}^nW_\mathrm{b}}$

其中,*t*_g_为梯度运行时间(min), *W*_b_为峰宽(min), *n*为峰数,*n*_c_为梯度洗脱维度峰容量。根据计算分析,梯度洗脱一维逆流色谱分离峰个数为18个,二维HPLC分离峰个数为53个,二维分离理论总峰容量达到954个。相比于单一维度的分离,全二维色谱分离大大提高了样品分离能力。

#### 2.3.2 空间覆盖率与线性相关性

空间覆盖率是指二维分离过程中溶质对二维分离空间的利用情况,是二维分离实际峰容量计算的重要参数。空间覆盖率有多种计算方法,常见的有格子计数法和凸包计算法,其中凸包计算法能够涵盖线性溶剂化能关系所预测的最大期望覆盖面积,不受格子计数法中格子个数、峰宽的影响,不需要考虑覆盖区域的具体形状,可采用Matlab脚本快速、准确的运行计算。实验结果表明采用凸包计算法得到的空间覆盖率为68.1%。

线性相关性是指被分离溶质在二维色谱分离过程中与两个维度固定相以及流动相之间的相互作用的差异性,主要体现了分离机制的差异性引起溶质保留行为的差异。本研究中逆流色谱与液相色谱尽管均采用反相洗脱模式,由于其分离机制的差异引起溶质保留行为的差异非常显著。当线性相关系数*r*为0时,说明两个维度分离具有很弱的相关性,两个维度色谱对溶质选择性的差异很大,即很强的正交性;当线性相关系数*r*为1时,说明两个维度分离具有很强的相关性,两个维度色谱对溶质选择具有很强的相似性,基本不存在正交性^[21]^。本文分别对逆流色谱与液相色谱各个出峰时间进行归一化处理,计算归一化处理后的数据相关性,得到*r*为0.17,表明两个维度的色谱分离莪术油具有弱相关性,即该二维色谱体系具有良好的正交性。进一步表明离线全二维逆流色谱-液相色谱是分离莪术油等复杂样品的一种有效方法。

## 3 结论

本文主要考察了全二维逆流色谱-液相色谱分离莪术油成分的能力,通过建立并优化各自的分离分析方法,构建了莪术油成分的二维色谱等高线图谱,并采用空间覆盖率、线性相关性以及峰容量等参数对其正交性进行定量分析。研究结果表明,与一维色谱分离相比,二维色谱峰容量提高了10倍以上。研究工作不仅为天然产物等复杂体系提供了新的分离分析方法,同时,由于流动相的良好兼容性优势,为构建新型的全二维色谱方法提供了思路。
